# Biomaterials and bioengineering to guide tissue morphogenesis in epithelial organoids

**DOI:** 10.3389/fbioe.2022.1038277

**Published:** 2022-11-17

**Authors:** Eun Young Jeon, Leila Sorrells, Hasan Erbil Abaci

**Affiliations:** ^1^ Dermatology Department, Columbia University Medical Center, New York, NY, United States; ^2^ Biomedical Engineering Department, Columbia University, New York, New York, United States

**Keywords:** morphogenesis, extracellular matrix, biomaterials, epithelial tissue, organoids, engineering

## Abstract

Organoids are self-organized and miniatured *in vitro* models of organs and recapitulate key aspects of organ architecture and function, leading to rapid progress in understanding tissue development and disease. However, current organoid culture systems lack accurate spatiotemporal control over biochemical and physical cues that occur during *in vivo* organogenesis and fail to recapitulate the complexity of organ development, causing the generation of immature organoids partially resembling tissues *in vivo*. Recent advances in biomaterials and microengineering technologies paved the way for better recapitulation of organ morphogenesis and the generation of anatomically-relevant organoids. For this, understanding the native ECM components and organization of a target organ is essential in providing rational design of extracellular scaffolds that support organoid growth and maturation similarly to the *in vivo* microenvironment. In this review, we focus on epithelial organoids that resemble the spatial distinct structure and function of organs lined with epithelial cells including intestine, skin, lung, liver, and kidney. We first discuss the ECM diversity and organization found in epithelial organs and provide an overview of developing hydrogel systems for epithelial organoid culture emphasizing their key parameters to determine cell fates. Finally, we review the recent advances in tissue engineering and microfabrication technologies including bioprinting and microfluidics to overcome the limitations of traditional organoid cultures. The integration of engineering methodologies with the organoid systems provides a novel approach for instructing organoid morphogenesis *via* precise spatiotemporal modulation of bioactive cues and the establishment of high-throughput screening platforms.

## 1 Introduction

Organ morphogenesis is guided by multiple interactions between cells and extracellular matrix (ECM) and a balance of exogenous cues in a spatiotemporally-controlled manner (Rezakhani et al., 2021). The ECM surrounding cells provides structural support for tissue integrity and tissue-specific biophysical and biochemical cues for cell adhesion, differentiation, and homeostasis as well as functions as a reservoir of growth factors and cytokines (Frantz et al., 2010). In mammals, there are two main types of ECM including the interstitial connective tissue matrix for physical support and the basement membrane separating epithelium from the connective tissue. The interstitial matrix is mainly composed of collagen type I and fibronectin, whereas the basement membrane is a dense sheet-like form mainly consisting of collagen IV, laminins, and proteoglycans (Bonnans et al., 2014). The components and organization of ECM vary depending on different tissues and temporal-specific remodeling such as tissue development and wound healing. Therefore, dysregulation of ECM remodeling including composition, structure, orientation, and stiffness leads to aberrant organ morphogenesis and pathological conditions, such as fibrosis and cancer (Tian et al., 2013; Bonnans et al., 2014).

Advances in our understanding of pluripotent stem cells (PSCs) and their capabilities to self-organize have led to the establishment of 3D culture systems that better mimic tissue-specific multiple cell types, structures and metabolic functions, called organoids (Fatehullah et al., 2016; de Souza, 2018). These organoid systems are a powerful tool to study human organ development and disease, enabling more realistic drug screening platforms than 2D cultures and discovery of new therapies (Broutier et al., 2017). To date, substantial efforts have been made to identify soluble signaling factors and modulators to control stem cell fate in organoid applications, and thus most of organoid cultures rely on a limited set of 3D supporting materials, mainly animal-derived Matrigel, which is an ill-defined heterogeneous basement membrane matrix (Yin et al., 2016). Despite its poorly-defined composition and the lack of tunability and reproducibility, researchers continue to use Matrigel because of its availability and a historical lack of comparable alternatives (Aisenbrey and Murphy, 2020). However, Matrigel cannot sufficiently and accurately induce distinct niche signals required for each organ morphogenesis. Therefore, engineering extracellular microenvironments with chemically and biophysically defined features can contribute to the establishment of more physiologically relevant organoid models that contain *in vivo*-like levels of cell phenotype diversity (Aisenbrey and Murphy, 2020; Kozlowski et al., 2021).

Despite unprecedented structural and functional resemblance to the corresponding organ, current reliance on uncontrolled, spontaneous morphogenesis that occurs within cell aggregates inherently results in the high variability of self-organizing growth and deficiencies in organoid anatomy and cellular composition, which hinders faithful experimental readouts (Yin et al., 2016; Marti-Figueroa and Ashton, 2017). In contrast, *in vivo* morphogenesis is instructed by external stimuli supplied in a precise spatial and temporal order (Lee et al., 2022). Recent breakthroughs have been achieved through the integration of existing tissue engineering methodologies that provide dynamic environments with biochemical, biophysical, and geometrical cues and allow better spatiotemporal control over those factors. These approaches include the use of chemically-defined engineered hydrogels for increasing the reproducibility and providing desirable properties, including topography, stiffness, degradability, and viscoelasticity (Gjorevski et al., 2016; Brassard et al., 2021). Moreover, the sophisticated design of microfluidic devices enables the creation of morphogen gradients within the organoid for controlled symmetry breaking (Manfrin et al., 2019), recapitulation of mechano-physical parameters, such as fluid flow, mechanical forces, and movements, (Kasendra et al., 2018; Jalili-Firoozinezhad et al., 2019; Shin and Kim, 2022) and vascularization (Homan et al., 2019).

In a mammalian body, most epithelial tissues contain multipotent stem cells that are responsible for tissue self-renewal and regeneration after damage (Slack, 2000). The range of their physiological functions is vast and involves the mechanical integrity of surfaces (skin), absorption (intestine), secretion of bioactive molecules (liver), filtration (kidney), and gas exchange (lung) (Gumbiner, 1992). Defining ECM components of each organ is important to understand their role in tissue development, function, repair, and disease. In the following section, we briefly summarize the composition, organization, and functions of ECM found in epithelial tissues including skin, intestines, kidney, pancreas, and lung. We also outline different types of hydrogels based on biomaterials ranging from naturally occurring materials to synthetic polymers that have been used for intestinal organoid culture. In particular, several well-established systematic studies provide key determinants for designing extracellular scaffolds, such as stiffness, degradability, and cell-binding motif that regulate cellular behaviors. Still, even with groundbreaking advanced in hydrogel fabrication techniques, these protocols recapitulate the early stages of *in vivo* organogenesis with partial accuracy, leading to the generation of developmentally immature tissues due to inability to exert spatial and temporal control over biochemical and physical factors (Marti-Figueroa and Ashton, 2017). In the main part, the integration of tissue engineering methodologies for instructing anatomically biomimetic morphogenesis will be discussed. Although these engineering approaches are generally proof-of-concept studies, they offer great possibilities to address main limitations of traditional organoids cultures.

## 2 Native ECM in adult epithelial tissues

### 2.1 Skin

The skin is a multilayered organ mainly consisting of the epidermis and the dermis, equipped with appendages, such as hair follicles and glands. The epidermis, the outermost layer of skin, is a stratified epithelium containing keratinocytes and is critical for protection the body from external stresses including pathogens and chemicals, hydration, and regulation of body temperature (Pfisterer et al., 2021). Within the interstitial matrix of the dermis, cells such as fibroblasts, immune cells, and vascular structures are in tight interaction with the ECM that provides structural support to these cells (Uitto et al., 1989). The primary component of skin ECM is known to be collagen, taking up an estimated 77% of the fat-free dry weight of human skin (Weinstein and Boucek, 1960). Collagen in the dermal matrix is composed of type I (80–85%) and type III (8–11%) collagens that are responsible for the tensile strength of skin (Davison-Kotler et al., 2019). Collagen type IV associated with laminin, fibronectin and other proteoglycans contributes to the formation of sheet-like basal lamina which epithelial cells attach to (Uitto et al., 1989). Elastin is thought to make up only around 4% of the fat-free dry weight of the dermis in skin, yet a network of elastic fibers contributes heavily to the elasticity, stretch, and recoil of skin (Hult and Goltz, 1965). Additionally, both fibrillin 1 and 2 proteins secreted by dermal fibroblasts and keratinocytes contribute to this elasticity and the creation of microfibrils in the dermal-epidermal junction (Haynes et al., 1997). Glycoproteins such as fibronectin and laminin, act as multi adhesive matrix proteins that provide anchor points for cell adhesion, migration, and proliferation (Bonnans et al., 2014; Pfisterer et al., 2021). Another central component is proteoglycans that are essential regulators of hydration, homeostasis, and wound healing. Prominent proteoglycans found in the skin are decorin and versican in the interstitial matrix and perlecan in the basement membrane (Bonnans et al., 2014).

### 2.2 Intestine

Intestinal epithelium is a representative self-renewal organ with extremely fast cellular turn-over rate. During this self-renewal process, Lgr-5+ intestinal stem cells located at the bottom of the crypt generate transit amplifying cells that migrate upwards until they reach the upper gland region and then differentiate into various cell types, such as enterocytes, goblet cells, enteroendocrine cells, and tuft cells *via* a number of cell division (Barker et al., 2008). The intestinal epithelium directly contacts with the surrounding basement membrane mainly composed of laminin, collagen IV, fibronectin, and proteoglycans for the maintenance of the intestinal microenvironment, stability, and regeneration (Beaulieu, 1997; Groulx et al., 2011).

In the intestine, composition and distribution of ECM vary depending on the anatomical location. For example, the reciprocal expression of different laminin isoforms was observed along the crypt-villus axis. In the human small intestine, laminin-111 and laminin-211 are spatially distributed at the upper crypt-villus and the lower crypt zone, respectively, while laminin-322 is restricted only to the villus (Beaulieu and Vachon, 1994). Similarly, in the human intestine, there are different collagen IV-α chains differentially distributed throughout the epithelial basement membrane. While α3(IV) and α 4(IV) chains are restricted on the top of the villus zone and α5(IV) and α6(IV) chains are expressed in the crypt region, α1(IV) and α2(IV) chains are detected along the entire crypt-villus axis (Simoneau et al., 1998; Sato et al., 2007). Fibronectin and its specific integrin receptor (α5β1) were found mostly confined in the crypt zone. Fibronectin has been confirmed to be associated with the regulation of intestinal crypt cell functions *via* argenin-glycin-aspartic acid (RGD) sequence-mediated interaction with epithelial cells (Benoit et al., 2012). Moreover, it has been shown that glycosaminoglycan molecules exert essential roles in retaining lubrication and structural integrity (Meran et al., 2017). Heparan sulfate found on the basolateral surface of intestinal epithelium has been reported to enhance intestinal regeneration by modulating Wnt/β-catenin signaling (Yamamoto et al., 2013). Moreover, some studies suggested that HA regulated intestinal stem cells (ISC)proliferation and intestinal growth including entire length, crypt depth, and villus height, through the binding to its various receptors (Riehl et al., 2012; Meran et al., 2017).

### 2.3 Liver

Liver has a highly organized and vascularized organ with many hepatic lobules as its functional unit. Each lobule has three different zones including a central vein, portal triad consisting of portal vein, hepatic artery, and biliary duct, and single sheets of hepatocytes that secrete bile salts to the bile duct (Gordillo et al., 2015). The liver lobule has attenuated ECMs, mainly consisting of collagen type I, III, IV, and V (Bedossa and Paradis, 2003). Collagen type I, III, and V are expressed in the portal tract and central vein wall, while in a sinusoid wall, collagen type IV takes part in the formation of a basement membrane-like low-density ECM required for rapid diffusion of molecules between the plasma and hepatocytes. The strategic position of ECM has been observed in a recent study about self-assembled human liver organoids. The organoids grown in acellular liver ECM scaffolds showed the presence of collagen I/fibronectin surrounding the hepatocytes and the localization of collagen IV/laminin around the developing bile duct structures (Vyas et al., 2018).

### 2.4 Pancreas

ECM composition differs in the peripheral and the interior of pancreatic islets, and collagen type IV and VI, and laminins are the most abundant ECM molecules (Stendahl et al., 2009). Collagen IV and VI are located at the islet-exocrine interface and basement membrane, and it was reported collagen IV significantly improved cell survival in human islets (Llacua et al., 2018). Laminins are found both in exocrine and endocrine parts of pancreas, but the spatial distribution of isoforms in islets are not well understood. In human islets, laminin-411 and laminin-511 are known to be essential for β-cell binding, proliferation and insulin transcription (Banerjee et al., 2012). Moreover, laminin-111 has been found to induce pancreatic ductal morphogenesis, possibly *via* an α6-containing integrin (Crisera et al., 2000). Fibronectin has also been found to be present in the human pancreatic epithelium and interacts closely with nidogen/entactin to create elasticity and a matrix connection (Uscanga et al., 1984). In addition, it was reported that RGD motif-containing ECM molecules, such as fibronectin and vitronectin, contributed to inhibition of apoptosis in mature human islets through RGD-dependent adhesion (Pinkse et al., 2006).

### 2.5 Kidney

The normal human kidney is made up of million filtering units called nephrons and is responsible for urine formation and blood filtration. In each nephron, there are three distinct compartments including glomerulus, a tubule, and vasculature (Bülow and Boor, 2019). The glomerulus for blood filtration contains the glomerular basement membrane (GBM) that provides adhesion for endothelial cells and podocytes and acts as a filtration barrier (Suh and Miner, 2013). The most essential components in both glomerular basement membrane and tubulointerstitial matrix are collagen type IV and laminin. During GBM assembly, initial laminin-111 and collagen IV α1α1α2 networks are sequentially replaced by laminin-511/521 and collagen IV α3α4α5 for long-term functional integrity (Abrahamson et al., 2013). Collagen IV in the mature GBM forms a mesh-like network, providing strength and structural stability (Suh and Miner, 2013).

### 2.6 Lung

Lung ECM is highly organized to serve many important functions including gas exchange and barrier protection. In the lung matrix, the most abundant ECM molecules are collagens, and various subtypes were found. Collagen IV is the major component of the very thin basement membrane separating alveolar epithelium and capillaries, providing structural stability and tensile strength (Burgess and Weckmann, 2012). Similarly, laminin was detected in the basement membrane of airway epithelium and became more tightly associated with alveolar capillary endothelial cells in mature lung (Luo et al., 2018). As another important ECM component, elastin is secreted by interstitial fibroblasts and widely distributed in most layers of airway wall, pulmonary blood vessels, and the parenchyma of the lung (Vindin et al., 2022). An interstitial matrix of elastin and collagen I between vascular cells contributes to viscoelasticity and strength for lung elastic recoil, preventing vessels from collapsing (Carmeliet, 2003). The lung also generates a provisional matrix consisting of fibronectin and fibrin during development and wound repair procedures (Burgess and Weckmann, 2012).

## 3 Biomaterials used in epithelial organoid applications

### 3.1 Matrigel

Matrigel is a basement membrane extract secreted by Engelbreth-Holm-Swarm mouse sarcoma cells and primarily consists of laminin, collagen IV, entactin, and heparin sulfate proteoglycan, and perlecan (Orkin et al., 1977). Matrigel has enabled the culturing of various organoid types including intestine (Sato et al., 2009), kidney (Zhong et al., 2014), liver (Hu et al., 2018), skin (Lee J. et al., 2018; Lee et al., 2020), and brain (Lancaster et al., 2013). However, with growing demands to precisely control organoid development, its uncharacterized components, batch-to-batch variation, and complexity hamper systematic studies. A recent proteomic study on Matrigel samples revealed significant proteomic heterogeneity within and among samples (Bi et al., 2020). In addition, the combinative use of fibrin hydrogel and 10% Matrigel resulted in the formation of early mouse intestinal organoids comparable to that of 100% Matrigel, which indicates that only certain Matrigel-induced signals are needed for initial organoid formation (Broguiere et al., 2018).

### 3.2 Collagen type I

**FIGURE 1:**
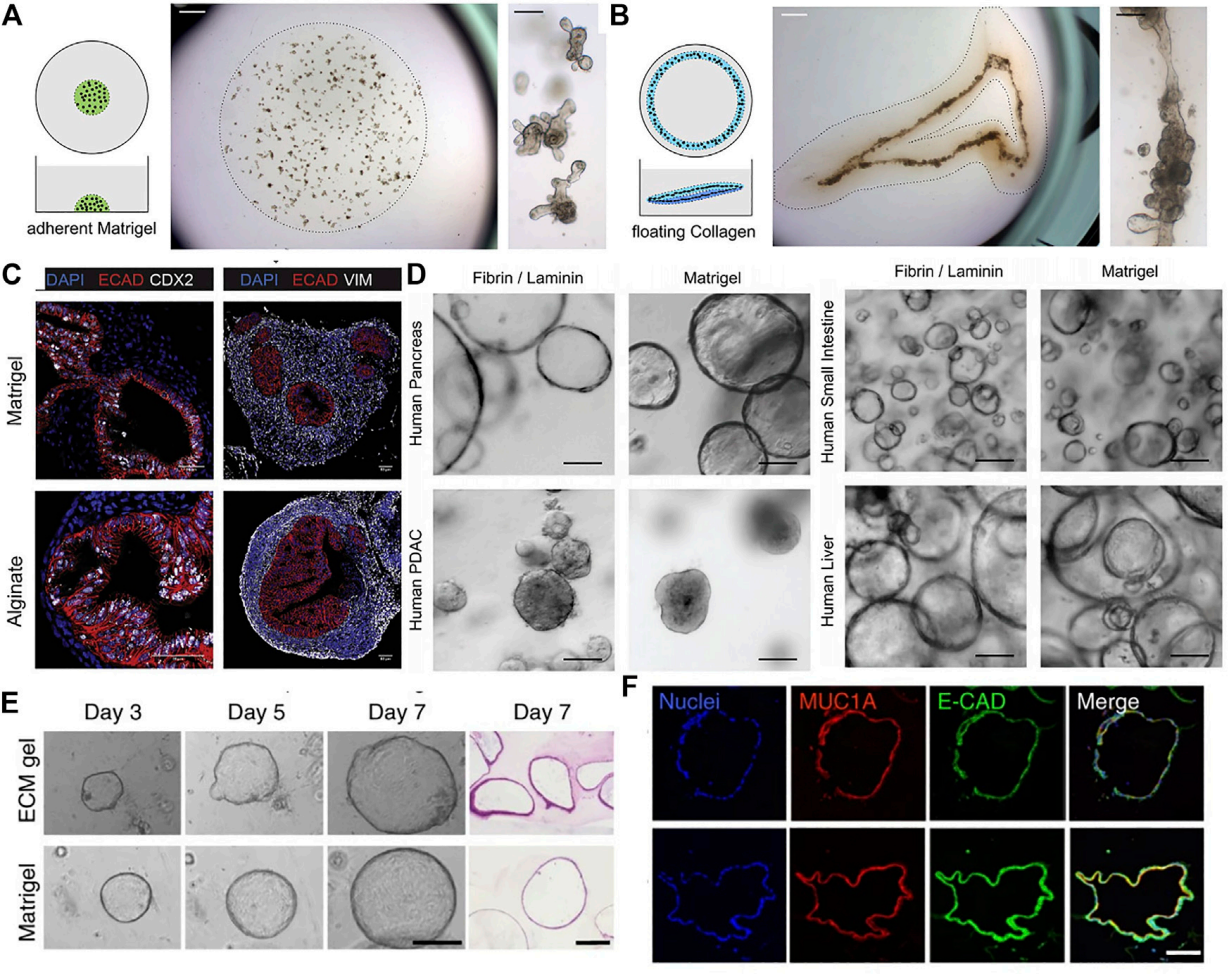
The use of different naturally-derived hydrogels for culturing epithelial organoids. **(A)** Traditional organoid culture in a plastic-adherent Matrigel. Mouse intestinal organoids grew as single budding cysts. **(B)** In vivo-like continuous tube formation of the intestinal organoids cultured in a floating contracting collagen hydrogel. Adapted from ref. (Sachs et al., 2017). **(C)** Immunostaining images of human intestinal organoids grown in alginate gel and Matrigel. Markers shown are ECAD for epithelium, CDX2 for intestinal epithelium, and VIM for mesenchyme. Adapted from (Capeling et al., 2019). **(D)** Bright-field images of various types of human epithelial organoids grown in fibrin hydrogel supplemented with laminin and in Matrigel. Adapted from ref. (Broguiere, et al., 2018). **(E)** Bright-field and H&E images of human pancreatic organoids cultured in porcine intestinal tissue-derived decellularized ECM hydrogel and Matrigel. **(F)** Immunofluorescence analysis of the pancreatic organoids showing expression of a pancreatic marker Mucin-1A and epithelial cadherin. Adapted from (Giobbe et al., 2019). Scale bars, **(C,F)** 50 μm **(A,B,D,E)** 100 μm, and **(A, B: right panels)** 1 mm.

Collagen type I is a highly abundant structural protein present in the interstitial connective tissue matrix of skin, tendon, cartilage, and ligament (Davison-Kotler et al., 2019). Collagen I has been widely used as a well-established simplistic 3D culture scaffold for culturing various cells and cell aggregates such as spheroid and organoids (Kozlowski et al., 2021). In a few studies for intestinal organoid culture, collagen I-based hydrogels have been shown to support survival and growth of intestinal organoids but display smooth appearance without or less crypt-like budding compared to the budding cysts grown in Matrigel (Jabaji et al., 2013; Jabaji et al., 2014; Jee et al., 2019). Collagen-based hydrogels have several advantages including simple composition and tunable physical properties, which are useful for manipulation of cellular differentiation and *in vitro* recapitulation of tissue morphogenesis (Mason et al., 2013; Carey et al., 2017). Indeed, a study reported that epithelial organoids cultured in a contracting floating collagen gel showed *in vivo*-like aligned and fused intestinal tube formation while traditional organoid cultures using Matrigel and adherent collagen gels did not support tube formation (Sachs et al., 2017) (Figure 1A,B). It is thought that the shrinking of floating collagen gel allowed organoids to physically align adjacent to one another. In a similar way, single primary human mammary epithelial cell cultured in floating collagen hydrogel grew as branched multicellular organoids (Buchmann et al., 2021). It was demonstrated that epithelial ductal elongation within the organoids relied on the mechanical response of the surrounding collagen network. During branch elongation, the invading epithelial cells caused tension that induced the macroscopic shrinkage of the collagen meshwork and then led to the formation of a mechanically stable cage that encased the organoid. Such matrix encasing in turn directed further branching morphogenesis. Another study took advantage of modularity of collagen hydrogel to determine key physical parameters for intestinal organoid functionality (DiMarco et al., 2014). The peristaltic contractility of the organoids was observed in collagen gels with a narrow optimal range of ∼22% porosity, a stiffness of ∼27 Pa, and a gel diameter equal to or less than 15.5 mm. Moreover, a collagen gel with an air-liquid interface provided long-term methodology for primary mouse intestinal culture (>360 days) by improved oxygenation of the organoids (Ootani et al., 2009). Recent efforts have been made to provide more actual tissue-relevant complex microenvironment through incorporation of other key factors required for tissue-specific morphogenesis into collagen I hydrogel (Sokol et al., 2016). While Matrigel or plain collagen I gel failed to induce a ductal growth of mammary epithelial cell-based organoids, the multicomponent gels including collagen I, laminin, fibronectin, and hyaluronic acid led to complex ductal and lobular morphologies resembling the epithelial structure of human breast.

### 3.3 Alginate

Alginate is an algae-derived polysaccharide and forms a non-covalent, electrostatically crosslinked hydrogel with controllable mechanical properties upon the addition of calcium chloride (Lee and Mooney, 2012). Capeling et al. utilized alginate as a 3D scaffold lacking inherent cell recognition to investigate the ability of human intestinal organoids containing mesenchymal and epithelial composition to create their own niche (Capeling et al., 2019). As a result, among five different alginate concentrations, 1% alginate scaffold could provide desired mechanical microenvironment for initial intestinal organoid formation, but the efficiency was significantly lower than when using Matrigel. Both alginate and Matrigel-grown intestinal organoids developed an inner epithelium surrounded by an outer mesenchyme in a similar manner (Figure 1C). However, when embedding primary human epithelial-only organoids (enteroids) into alginate hydrogels, the alginate did not support organoid expansion due to the absence of an exogenous ECM, while enteroids grown in Matrigel were highly proliferative, suggesting that mesenchyme is essential in forming a niche to promote epithelial proliferation in the bioinert alginate gel.

### 3.4 Fibrin

Fibrin, an ECM protein derived from the cleavage of fibrinogen is widely utilized in the field of tissue engineering (Moesson, 2005) (Zisch et al., 2001). The use of fibrin-based hydrogel has been also reported in epithelial organoid culture. Broguiere and colleagues found that fibrin gel could provide an appropriate physical support for organoid cultures at the optimal concentrations between 3 and 4.5 mg/ml, however, the supplementation of laminin-111 is crucial for the formation, long-term expansion, and crypt-like budding of both mouse and human epithelial organoids (Broguiere et al., 2018). Moreover, the fibrin/laminin hydrogel was also applicable to expansion of other human epithelial organoids including liver, pancreas, and pancreatic ductal adenocaricinoma (PDAC) (Figure 1D). Fibrin crosslinking was also utilized to fabricate hiPSC-derived hepatic cells-laden composite hydrogel capsules with additional crosslinking of alginate and chitosan (Wang et al., 2020). The encapsulated hepatic cells were self-organized into liver organoids and showed markedly higher expression of hepatocyte and cholangiocyte-related genes compared to those cultured in the capsules without fibrin. Liver-specific markers and functions. The liver organoids also showed liver-specific functions including urea synthesis and albumin secretion.

### 3.5 Decellularized ECM

Similar to Matrigel, dECM has heterogenous composition derived from original tissue ECM. After decellularization, the preserved tissue-specific ECM greatly affects cell morphology and behaviors including proliferation, differentiation, and migration due to their inherent biochemical cell-instructive characteristics (Zhang et al., 2009). A variety of dECM derived from other tissues have been developed for tissue engineering applications since 1970s. By incorporating organoids, dECM-based hydrogels have been employed as exogeneous platforms, which can be applied to regenerative medicine applications with patient-derived organoids.

In a recent study, a porcine intestinal tissue-derived dECM gel was designed to support *in vitro* organoid cultures (Giobbe et al., 2019). Proteomic analysis revealed that the ECM protein composition of the hydrogel showed similarities with endoderm tissue. The dECM hydrogels supported the formation and growth of endoderm-derived organoids including small intestine, liver, pancreas, and stomach (Figure 1E,F). Furthermore, human fetal pancreatic organoids and small intestinal organoids grown in the dECM gel were transplanted subcutaneously in immunodeficient mice, respectively. The organoids survived, maintaining their organization and signature expression at protein level for several weeks with no major differences between dECM gel and Matrigel. Moreover, Spence and colleagues reported a protocol for the generation of lung organoids using a decellularized human lung matrix. Lung organoids seeded in the dECM hydrogels gave rise to epithelial structures with expression of multiciliated-cell markers after 40 days of culture (Miller et al., 2019). Another study also demonstrated that when hPSC-derived foregut spheroids were seeded on slices of decellularized human lung matrix, they developed into human lung organoids with fully matured muliciliated cells and proximal airway-like structures that were not observed in Matrigel or media rich in fibroblast growth factor 10 (FGF10) (Dye et al., 2015).

### 3.6 GelMA

Gelatin methacryloyl (GelMA) is a modified gelatin containing a majority of methacrylamide groups and a minority of methacrylate groups, which can be crosslinked through photopolymerization (Yue et al., 2015). GelMA has been widely utilized to form an organized vascular-like structures due to its instant crosslinking ability, tunable mechanical properties, and biocompatibility (Nie et al., 2018). A GelMa-based sacrificial bioprinting technique was developed to fabricate vascularized networks in a hydrogel construct. The hydrogel with microchannels supported viability of the encapsulated MC3T3 cells for 7 days due to better mass transport, whereas the hydrogel without microchannels showed 60% cell viability (Bertassoni et al., 2014). Moreover, the perfusion of endothelial cells led to the formation of endothelial lining within the fabricated channels. In another case, a perfusable 3D hepatic construct was fabricated by direct bioprinting of HepG2/C3A spheroids-laden GelMA (Bhise et al., 2016). The vascularized liver model remained hepatic functionality for 30 days as evidenced by the levels of key hepatic markers and provided a proof-of-concept demonstration of the utility for hepatotoxicity evaluation of a drug. GelMA also serves as a bioprinting ink for precise printing of tissue analogs such as organoids. For example, bovine colon organoids pre-differentiated in Matrigel were transferred and embedded in 7.5% GelMA (w/v) supplemented with 0.1 mg/ml Matrigel for organoid bioprinting into 96 well culture plates. Live/dead staining showed that the printed colon organoids remained viable and proliferative for 48 h post-print (Töpfer et al., 2019).

### 3.7 Polyethylene glycol

PEG is the most common polymer of numerous synthetic hydrogels for organoid cultures due to its hydrophilic and biocompatible properties (Zhu, 2010). In addition, PEG is manufactured in a broad range of molecular weights and structures. Furthermore, PEG can be functionalized with reactive terminal groups, such as acrylates, thiols and NHS esters, which allows various crosslinking strategies, control of physical properties and degradation, and the incorporation of bioactive molecules (Zhu, 2010). Importantly, the modular nature of synthetic polymer-based systems offers great opportunities to study an interplay between the mechanical properties of matrix and biochemical signals in dictating stem cell differentiation (Engler et al., 2006; Musah et al., 2014).

**FIGURE 2:**
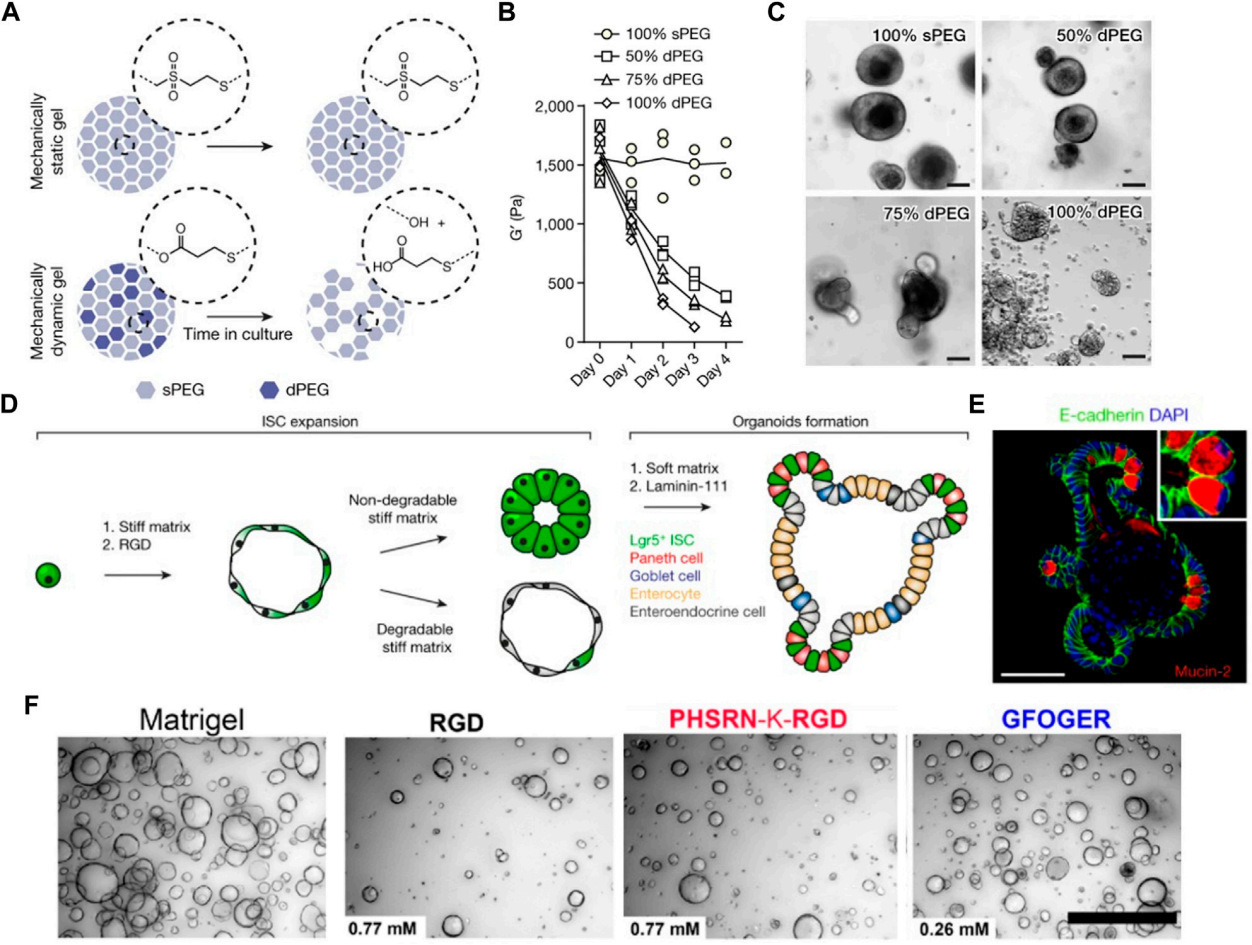
Intestinal organoid formation within chemically-defined engineered PEG hydrogels. **(A)** Schematic illustration of mechanically dynamic PEG gel with hydrolytically degradable crosslinks for matrix softening. **(B)** Mechanical characterization of hybrid PEG gels. **(C)** Organoid formation of mouse ISCs grown in matrices of different softening rates. **(D)** Schematic showing different stages of intestinal organoid formation within the engineered PEG hydrogel. **(E)** Immunofluorescence staining of intestinal organoid containing differentiated intestinal cells. Adapted from ref. (Gjorevski et al., 2016). **(F)** Bright-field images of the formation of human intestinal enteroids cultured in Matrigel and different PEG gels containing RGD, PHSRN-K-RGD, and GFOGER peptides. Adapted from ref. (Hernandez-Gordillo et al., 2020). Scale bars, **(A,B,E)** 50 µm and **(F)** 500 µm.

A fully defined, synthetic hydrogel based on 4-armed PEG conjugated with adhesive peptides was designed to support the formation and expansion of hESC- and hiPSC-derived intestinal organoids (Cruz-Acuña et al., 2017; Cruz-Acuña et al., 2018). They investigated the effects of polymer concentrations and adhesive peptide types on cell viability and intestinal morphogenesis. Organoids grown in 4% PEG hydrogel functionalized with RGD peptides maintained the highest viability for 7 days after encapsulation. Similarly, another study demonstrated the incorporation of different ECM molecules such as fibronectin, laminin, and collagen type IV into enzymatically crosslinked PEG hydrogels supported ISC survival and growth, while inert PEG hydrogels failed (Gjorevski et al., 2016; Gjorevski and Lutolf, 2017) (Figure 2A). In particular, an optimal stiffness (∼1.3 kPa) supported ISC expansion through a yes-associated protein 1 (YAP)-dependent mechanism (Figure 2B). On the other hand, a softer matrix and laminin-based adhesion improved ISC differentiation and intestinal organoid formation. Based on these findings, they designed enzymatically degradable PEG hydrogels containing laminin-111 that could undergo matrix softening over time through hydrolytic degradation supported intestinal morphogenesis (Figure 2C,D,E). The softening profile of the gels was easily tuned by adjusting the mixing ratio of non-degradable and degradable PEG polymers. A recent study also leveraged the modularity of PEG-based hydrogel to investigate the key parameters required for human intestinal organoids (Hernandez-Gordillo et al., 2020). Integrin-binding ligands such as collagen-derived peptide (GFOGER) and fibronectin-derived peptide (PHSRN-K-RGD) were grafted to 8-arm PEG gels containing MMP-cleavable crosslinkers. They identified that the presence of GFOGER peptide supported the growth and formation of human intestinal enteroids and endometrial organoids (Figure 2F).

Similar to intestinal organoids, other organoid types have been also generated in PEG-based hydrogels. Enzymatically crosslinked inert PEG hydrogel was engineered to mimic the physiological stiffness of the liver (Sorrentino et al., 2020). Then, the supplementation of key ECM proteins found in the liver, such as fibronectin and laminin-111 or ECM-derived RGD peptide led to efficient generation of liver organoids, comparable to Matrigel. In pancreatic organoid culture, it was observed that embryonic pancreatic progenitors could be maintained and expanded in PEG-based hydrogels functionalized with laminin-111, while non-functionalized hydrogel induced the progressive loss of pancreatic and epithelial phenotye (Greggio et al., 2013). Moreover, in comparison with stiffer gels (G’>1 kPa), only soft gels with a shear modulus (G’) of ∼250 Pa were able to sustain cluster formation and progenitor maintenance. It has been further demonstrated that PEG-based hydrogels serve as a tunable platform for the systematic investigation of the role of mechanical cues in epithelial morphogenesis of kidney cells (Weber et al., 2017). Among three hydrogel variants of degradable PEG, non-degradable PEG-heparin, and degradable PEG-heparin gels, the soft MMP-susceptible hydrogel consisting of both starPEG and heparin was found to be induce the morphogenesis of renal proximal tubule epithelial cells into physiologically-relevant polarized tubule structures. They also demonstrated the applicability of the established renal tubulogenesis model for nephrotoxicity testing.

In summary, engineering biomimetic ECM is key for stem cell self-renewal and differentiation. Although the use of nature biomaterials, such as Matrigel and collagen type I, are often preferred due to their abilities to support cell adhesion and regulate various cellular process including differentiation into specific lineages, the organoids often showed highly heterogeneous morphology and variability in maturity and functionality. Alternatively, synthetic biomaterials are often inert and can be easily manipulated to establish tunable and highly reproducible cell-instructive microenvironments. Based on characteristics of native organ ECM, elaborately designed advanced matrices would provide more faithful and improved control over the growth and differentiation of organoids.

## 4 Engineering approaches to instruct organoid morphogenesis

### 4.1 Engineered hydrogel-based scaffolds

*In vivo* native tissues and organs are characterized by various morphologies and topographies to present tissue-specific functions. Microengineered scaffolds with defined geometries, including surface topologies and internal structures, can efficiently guide stem cell self-organization and patterning towards high-order functional structures that resemble the target tissue. In 2006, it was reported that the initial geometry of hydrogels dictated the position of branching and new tubules formation of mammary epithelial cells grown within the scaffold (Nelson et al., 2006). This process was determined by autocrine inhibitory morphogens secreted locally by the epithelial cells.

**FIGURE 3:**
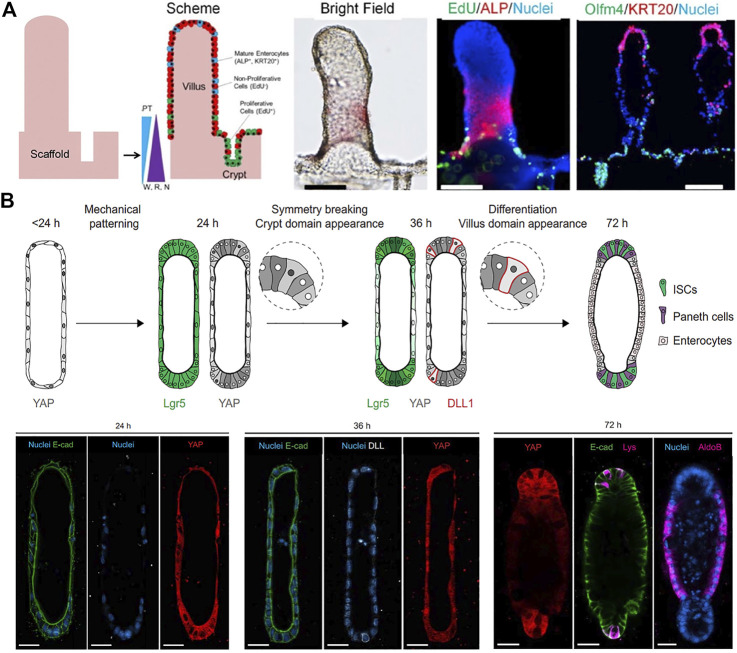
Geometry-guided morphogenesis of intestinal organoids. **(A)** *In vitro* crypt-villus structure formation and tissue polarization by culturing human intestinal cells on a micropatterned collagen scaffold under the gradient of growth factors (W: Wnt-3A; R: R-spondin 3; N: noggin). Adapted from ref. (Wang et al., 2017). **(B)** Schematic illustration of the proposed mechanism of the initial geometry-driven epithelial organoid patterning. Immunofluorescence images showing the distribution of YAP cells, Notch ligand DLL + cells, enterocytes (aldoB), and Paneth cells (Lys) in the organoids at 24, 36, and 72 h of ISC seeding. Adapted from ref. (Gjorevski et al., 2022). Scale bars, **(A)** 100 μm and **(B)** 25 μm.

In intestinal organoids, crypt-villus structures are formed at random orientations and in variable numbers. Microstructured scaffolds as artificial geometrical cues allow spatially controlled crypt patterning of intestinal organoids in an identical manner, predicting the number and location of crypt domains. Culture of human intestinal cells on a micropatterned collagen scaffold mimicking topography of the intestine generated *in vitro* self-renewing human small intestinal epithelium with a crypt-villus architecture, and open and accessible luminal surfaces (Figure 3A) (Wang et al., 2017). These shape-guided human intestinal tissue then displayed a polarized crypt-villus unit under the gradients of three growth factors (Wnt3, R-spondin, and Noggin) and the opposing gradient of APT, a gamma secretase inhibitor. A similar approach to engineer macroscopic intestinal surfaces also recreated crypts of intestines by applying chemical gradients to the established epithelium onto micromold collagen scaffold (Wang et al., 2018). This construct showed compartmentalization of proliferative and differentiated cells and migration replicating crypt biology. However, these attempts relied on complex chemical gradients and have not recapitulated multicellular organization.

Alternatively, in 3D hydrogel composed of type I collagen and Matrigel within tubular-shaped microcavities, ISC grew into an intestinal organoid with characteristic crypt and villus structure along predefined spatial boundaries (Nikolaev et al., 2020). Most importantly, the organoids showed long-term homeostasis due to the open-ended accessible tubular structures and continuous removal of dead cells in microfluidic culture conditions. The intestinal organoids showed the emergence of specialized cell types that were rare in conventional organoids. The same group studied the underlying mechanism of how the initial geometry dictated epithelial patterning and morphogenesis (Gjorevski et al., 2022). Their data suggested that geometry-driven patterning resulted from spatial differences in cell packing and heterogeneities in yes-associated protein (YAP1) mechanosensing/transduction and Notch signaling, which in turn specify crypt or villus domain (Figure 5B). The cells at the ends became more packed due to crowding within a limited space, whereas cells in the lateral regions remained spread. Measurement of YAP activity throughout the tissue demonstrated that tissue geometry could control the spatially patterned activation of YAP and localization of Paneth cell differentiation through differential cell spreading.

Furthermore, 3D bioprinting of cell aggregates makes it possible for manufacturing organ-specific tissues with the desired microarchitecture, cellular density, composition, and function. Aspiration-assisted freeform bioprinting enabled precise positioning and direct fusion of stem cell-derived spheroids within a self-healing support gel and built a variety of constructs with different configurations (Ayan et al., 2020; Daly et al., 2021; Kim et al., 2022) Aspiration force was employed to reduce the spheroid damage and premature assembly that often occur in the conventional extrusion bioprinting. Moreover, in a study that utilized an embedded 3D printing method, iPSC-derived organoids were compacted *via* centrifugation to form a living tissue matrix, and a sacrificial gelatin ink was printed within the matrix that serves as perfusable channels for custom-shaped vascularization in the pattern of single or branching conduit after removal of inks (Skylar-Scott et al., 2019). Using this approach, the living matrix composed of iPSC-derived cardiomyocytes and primary cardiac fibroblast created a functional and perfusable cardiac tissue that could fuse and beat synchronously over 7 days. More recently, another 3D bioprinting-assisted method was reported to establish intestinal epithelial tubes with a spontaneous morphogenesis process (Brassard et al., 2021). This allows direct printing and deposition of organoid-forming ISCs as building blocks into Matrigel/collagen type I matrices, controlling cellular aggregate density and tissue geometry. It was also found that co-decomposition of supportive cells, such as mesenchymal cells modulated morphogenesis in space and time by altering the initial phenotype of the self-organizing ISCs, improving lumen formation.

### 4.2 Spatiotemporal regulation of morphogen gradients

*In vivo* organogenesis is driven by not only intrinsic self-organizing ability of stem cells, but also external morphogen gradients created in a precise spatial and temporal order (Lee et al., 2022). Indeed, Hans Clevers’ group has reported the presence of Wnt3 gradients within the crypt of intestinal organoids derived from mouse cells (Farin et al., 2016). However, in traditional organoid culture systems, free floating EBs or EBs embedded in isotropic matrices are exposed to radially-symmetric biochemical niche signals and maintained under a static condition, where there is no control over the orientation of the resultant organoids. Conventional organoid systems also lack biomechanical control of the microenvironment. In contrast, the use of advanced microengineering methods, such as microfluidic devices and microparticles, allows us to extrinsically control the intrinsic capacity of stem cells for self-organization by providing dynamic microenvironments that can guide cellular and tissue organization.

#### 4.2.1 Microfluidic devices

Microfluidic technology can provide fine control over the spatial and temporal arrangement of multiple signaling molecules, which enables patterning of the cells exposed to chemical gradients (Bhatia and Ingber, 2014). For example, to recapitulate the development of epiblast and amniotic ectoderm, single hESCs were injected into the channel of a microfluidic device and subsequently clustered in gel pockets, where they grew into epiblast-like cysts (Zheng et al., 2019). At 36 h after addition of BMP4, cysts differentiated into amniotic ectoderm-like cells (AMLC) at the pole exposed to BMP4 and epiblast-like epithelium at the opposite pole, resembling a human bipolar embryonic sac before its gastrulation. Furthermore, the addition of IWP2, an inhibitor of Wnt-ligand secretion, and noggin, a BMP inhibitor, led to a more organized epiblast-like pole with AMLC patterning at the other pole, mimicking an anteriorized embryonic sac. Similarly, a microfluidic system was also designed to induce asymmetric cell fate patterning within 2D hiPSC colonies in response to a localized source of BMP4 and investigate the effects of morphogen concentration and cell density on the germ layer patterning (Manfrin et al., 2019). At higher BMP4 concentration, CDX2^+^ trophectoderm was mainly detected on BMP4 source side, which was rarely identified at lower concentrations. Exposing high density colonies to the gradients of BMP4 and opposing noggin resulted in robust restriction of mesoderm (MIXL1), endoderm (SOX17) and trophectoderm (CDX2) exclusively to BMP4 source side and the expression of SOX2 to the noggin side.

Microfluidic systems also allow application of fluid flow and mechanical cues similar to those observed *in vivo*, which enhances organ-specific responses and functions. Dr. Ingber’s group reported a lung-on-a chip that recapitulated lung-specific breathing movements by controlling the inflow and outflow of air in the flexible side chambers (Huh et al., 2010). This microfluidic chip has been commercialized and been also utilized to establish gut-on-a chips involving peristalsis-like motion and flow (Kim et al., 2012; Kasendra et al., 2018). Similarly, another study reported the induction of 3D morphogenesis of an intestinal epithelium from organoid-derived epithelial cells within microfluidic devices that can offer continuous basolateral flow to remove morphogen antagonists (Figure 5A) (Shin and Kim, 2022). They developed two different culture platforms: a gut-on-a-chip with two convoluted microchannels and an elastic porous membrane in the middle, or a single channel microfluidic hybrid chip with a Transwell insert. In both platforms, 3D intestinal morphogenesis occurred in 5 days after the flow was initiated.

Particularly, fluid flow is considered as a key parameter for induction and improvement of vascularization of organoids cultured in microfluidic systems. For this purpose, endothelial cells are co-cultured with organoids in a self-organizing method. Culturing hPSC-derived kidney organoids in a 3D printed microfluidic chip under high fluid flow induced substantial vascularization of glomerular region and morphological maturation compared with that in static controls (Figure 5B) (Homan et al., 2019). In addition, shear stress on hPSC-based retinal organoids grown in a microfluidic chip enhanced vascularization that is essential for photoreception of eye functions and led to maturation and polarization of retinal pigment epithelium (Achberger et al., 2019). This *in vitro* retinal model also demonstrated applicability for drug toxicity testing, as evidenced by the apoptosis of retinopathic cells under the addition of chloroquine and gentamicin. Furthermore, when hPSC-derived stomach organoids integrated into the microfluidic devices were subjected to peristaltic luminal flow and stretching, they demonstrated the ability to deliver fluorescent molecules into gastric lumen *via* peristaltic-like motility (Lee K. K. et al., 2018).

Efforts have been also made to emulate other external cues, such as oxygen level, for well-controlled morphogenesis (Shah et al., 2016; Jalili-Firoozinezhad et al., 2019). In human intestinal tissue, oxygen level is lower than in the air, which provides a sophisticated microenvironment for anaerobic microbial growth (Zheng et al., 2015). For example, to establish a physiologically relevant oxygen gradient across human intestinal epithelium and microvascular endothelium, a microscale oxygen sensor was integrated into the aforementioned chip developed by Dr. Ingber’s group, and the device was placed in an engineered anaerobic chamber (Figure 5C) (Jalili-Firoozinezhad et al., 2019). When compared to aerobically cultured chips, generation of oxygen gradient on the chip improved intestinal barrier function and sustained more than 200 different living bacterial species including both aerobic and anaerobic bacteria with similar complexity to a human stool microbiome. Another study reported that the ability of a microfabricated device to control oxygen supply across the entire organoids led to enhanced long-term organoid viability with decreasing necrotic tissue at the core (Berger et al., 2018).

**FIGURE 4:**
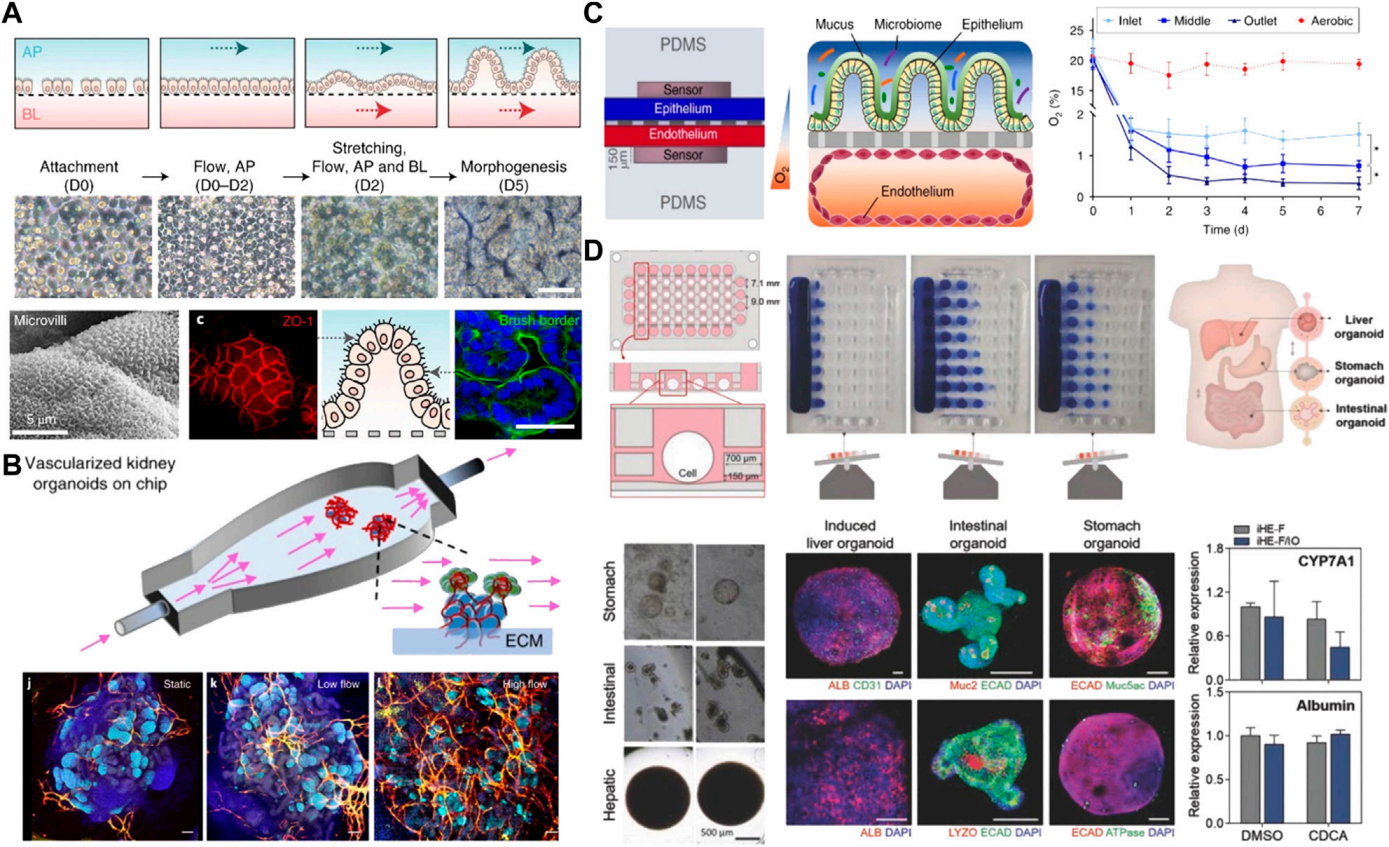
Advanced microfluidic-based platforms for mimicking better morphogenesis and the establishment of a multiorgan model. **(A)** Establishment of Intestinal morphogenesis in a gut-on-a-chip under both apical flow and basolateral flow and mechanical stretching after 5 days of culture. The culture epithelium showed the microvilli architecture and the formation of tight junctions. Adapted from ref. (Shin and Kim, 2022). **(B)** Vascularized kidney organoids culture within a perfusable chip under fluid flow and the influence of different flow conditions including static, low and high flow on vascularization. Adapted from ref. (Homan et al., 2019). **(C)** Two-channels intestine-on-a-chip with an oxygen gradient and oxygen concentration profiles between an aerobically-cultured endothelium channel and an anaerobically-cultured epithelium channel. Adapted from ref. (Jalili-Firoozinezhad et al., 2019). **(D)** A microfluidic-based multiorgan model of liver, small intestine, and stomach organoids in a microplate array format on the rocking culture system. Immunostaining analysis of specific markers for three different tissues and valid interorgan interaction evidenced by monitoring the expression of CYP7A1 and albumin in hepatic tissue. Adapted from ref. (Jin et al., 2018). Scale bars, **(A,B)** 100 μm and **(D)** 200 and 500 µm.

Sophisticated design of microfluidic devices allows us to build a multi-organ model that can communicate *via* fluid flow in a compartmentalized manner (Skardal et al., 2017; Jin et al., 2018; Skardal et al., 2020). In a liver-intestine-stomach multi-organoid system, the presence of media flow contributed to the formation of vascularized liver organoids composed of induced hepatic cells and decellularized liver ECM, and then the vascularized liver organoids were co-cultured with both mouse stem cell-derived intestinal and stomach organoids *via* fluidic interconnection between separated chambers (Figure 4D). Interorgan interaction was demonstrated by monitoring the expression of a bile acid-regulated enzyme CYP7A1 in hepatic tissue. Supply of bile acid to liver- and intestinal organoids led to lower level of CYP7A1 due to the secretion of paracrine factors from intestinal organoids, while the single culture of liver organoids maintained a consistent level of CYP7A1 (Jin et al., 2018).

#### 4.2.2 Microparticles

Microparticles can also create internal chemical gradients by releasing signaling molecules in a localized and controlled manner or sequestering morphogens (Carpenedo et al., 2009; Suri et al., 2013; Dang et al., 2016; Ben-Reuven and Reiner, 2020). McDevitt group firstly used co-aggregation of degradable poly (lactic-co-glycolic acid) (PLGA) microspheres containing retinoic acid and mouse ESCs to demonstrate the potency of controlled and localized delivery of a single morphogen within an EB (Carpenedo et al., 2009). Compared to direct treatment of soluble retinoic acid, EBs containing microspheres showed the formation of cystic spheroids and the spatial organization of the epiblast and visceral endoderm cell populations, which resembled E6.75 mouse embryos with an exterior visceral endoderm and an epiblast cell layer. In another study, consecutive loading and fusion of mESC-derived EBs containing BMP4-loaded gelatin microparticles and blank EB were achieved by a microfluidic-based trap array (Suri et al., 2013). The merged EB displayed patterning with spatially-controlled expression of a maker for primitive streak development and mesoderm differentiation which is analogous to the initiation of gastrulation in mouse embryos at E6.5.

### 4.3 Guided initial cellular assembly

Spatial orientation of cells and heterogeneous cellular compositions within an aggregate can instruct divergent cell fate, patterning, and morphogenesis. James Wells’ group recapitulated normal intestinal enteric nervous system (ENS) development by combining hPSC-derived neural crest cells (NCCs) with human intestinal organoids (HIO) (Workman et al., 2017). Intrinsic cues within HIO instructed NCCs to differentiate into neurons and glial cells of the ENS, and after engraftment *in vivo*, NCCs formed complex ganglionic structure similar to the embryonic development of myenteric and submucosal neural plexuses. These results suggest that controlling cellular composition during initial cellular assembly provides intrinsic information for subsequent morphogenesis through juxtacrine and paracrine signaling between neighboring cells (Ashton et al., 2012). Many other studies also support the need of cellular heterogeneity in the organoid system for proper tissue growth and development (Leeman et al., 2019; Jardé et al., 2020). In the intestinal organoids, incorporation of a mesenchymal niche enhanced gut growth and development *via* facilitated mesenchymal-epithelial crosstalk (Loe et al., 2021).

**FIGURE 5:**
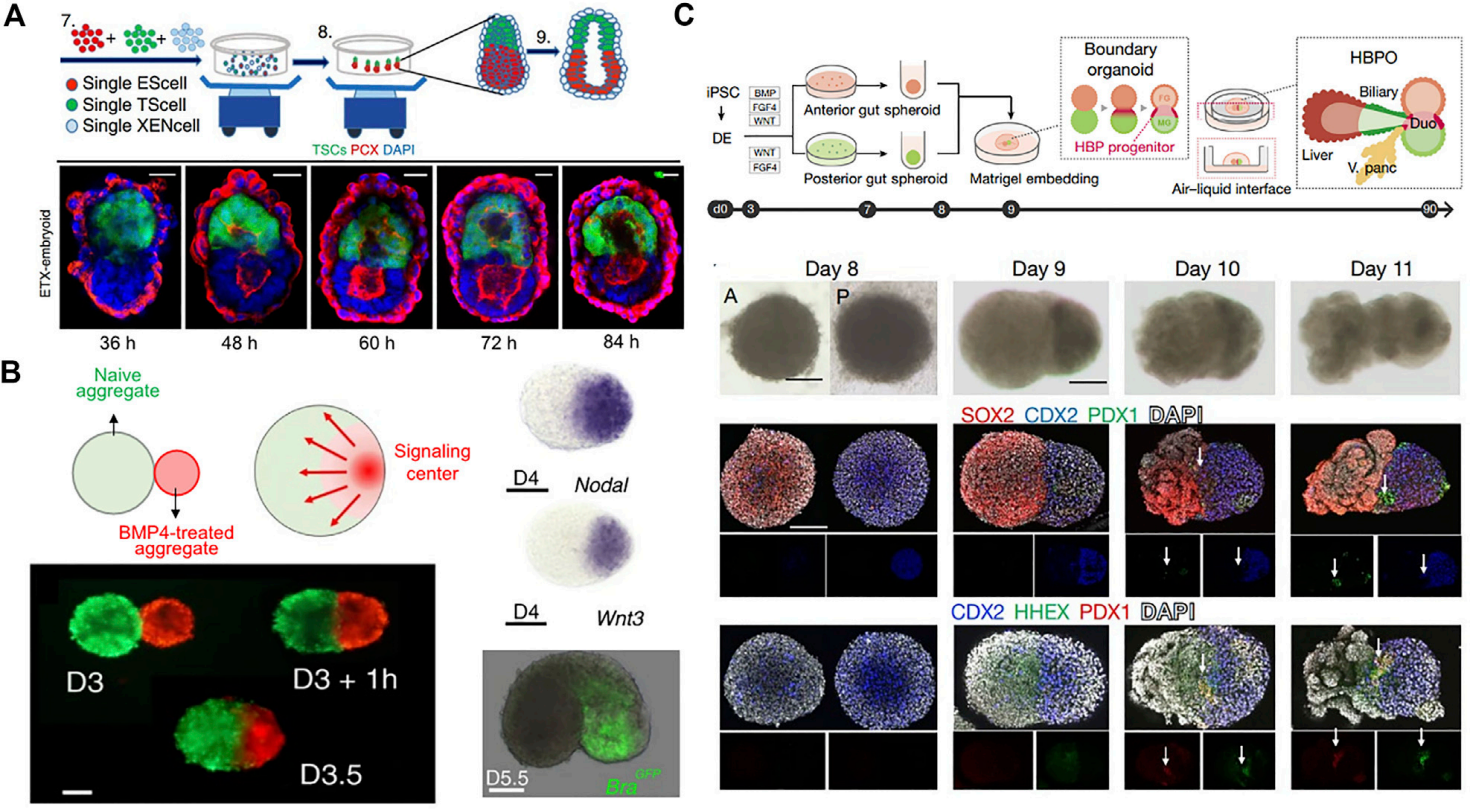
Guided morphogenesis by initial cellular assembly. **(A)** Schematic overview of generation of self-assembled ETX-embryoids from a combination of ESCs, TSCs, and XENCs and immuno-stained images showing the progression of pro-amniotic cavity formation. Adapted from ref. (Zhang et al., 2019). **(B)** BMP4-instructred (red) and untreated mESC aggregates (green) merged spontaneously forming an embryoid. BMP4 activation led to restricted expression of Wnt3 and Nodal in the signaling center, which induced expression of the mesoderm and primitive streak marker, brachyury (Bra). Adapted from ref. (Xu et al., 2021). **(C)** Schematic overview of generation of HBP organoid by merging hiPSC-derived anterior and posterior gut spheroids. Bright-field and whole-mount immuno-stained images for SOX2 (anterior cut cells), CDX2 (posterior gut cells), PDX1 (pancreas progenitor), and HHEX (early hepatic marker). HHEX and PDX1 were detected only at the boundary of the fused spheroids. Adapted from ref. (Koike et al., 2019). Scale bars, **(A)** 20 μm and **(B,C)** 100 µm.

Some developmental *in vitro* models to mimic morphogenesis have been investigated to recapitulate early embryonic self-organization and architecture. Self-assembly of mESC and mouse trophoblast stem cells (mTSCs) cultured in 3D scaffold of Matrigel developed into an elongated cylindrical architecture with a central pro-amniotic cavity similar to the post-implantation mouse embryo (Harrison et al., 2017). These embryos spontaneously initiated asymmetric and regionalized expression of mesoderm and primordial germ cell markers. However, this ETC-embryos lack primitive endoderm-derived cells failed to gastrulate. Another study incorporated extra-embryonic endoderm stem cells (XENCs) into this ETS-embryo model using a nonadherent suspension-shaking system (Figure 5A) (Zhang et al., 2019). The self-assembled ETX-embryoids exhibited lumenogenesis, asymmetric patterns of mesoderm and primordial germ cell precursors, and formation of anterior visceral endoderm cells, similar to an embryo at mid-gastrulation. More recently, engineering a localized morphogen signaling center within an aggregate guided morphogenesis and patterning by spontaneous breaking of symmetry (Figure 5B). A BMP4-treated mESC aggregate was merged with a larger untreated aggregate (Xu et al., 2021). Incubation of the aggregate with soluble BMP4 for 8 h resulted in inducting expression of Wnt and Nodal, key morphogens for gastrulation. BMP4-instructed aggregates thereby served as morphogen signaling centers that were asymmetrically located at one tip of the growing embryoids and allowed for the initiation of developmental programs. They demonstrated the formation of all three germ layers including endoderm, mesoderm, and ectoderm through a gastrulation process similar to a neurula-stage mouse embryo.

Strategies employing the fusion and coculture of multiple organoids have recently also emerged (Xiang et al., 2017; Koike et al., 2019). Fusion of hiPSC-derived anterior and posterior gut spheroids enabled retinoic acid-dependent formation of hepato-biliary-pancreatic (HBP) organ domains at the foregut-midgut boundary region in the absence of extrinsic factors (Figure 5C) (Koike et al., 2019). RNA sequencing analysis also revealed that anterior or posterior regions gained foregut or midgut-hindgut identity, respectively. However, *in vivo* transplantation of the fused spheroids into immunodeficient mice mainly developed into intestinal tissue with negligible expression of HBP markers. Nevertheless, the *in vitro* multiorgan integrated model provides an opportunity to study communication between gastrointestinal tract, liver, and pancreas.

Taken together, engineering approaches towards more physiologically-relevant organoids aim to recapitulate biochemical, mechanical, and functional characteristics of human organs and better understand organogenesis. From their findings, it is believed that organoid development is critically guided by extrinsic niche including *in vivo*-like spatiotemporally controlled morphogen gradients and tissue geometry as well as various systemic parameters including organ-like physical movements, fluid low, and oxygen level. Additionally, co-aggregation of multiple cell types can increase the complexity of *in vitro* morphogenesis by induction of spatiotemporal tissue-tissue interactions.

## 5 Conclusion and outlook

Organoids are powerful tools to recapitulate cellular diversity, structure, and function of native organs. However, current organoid culture systems rely on cell-intrinsic self-organization but lack precise control of external biochemical and physical cues to guide organ morphogenesis. Our understanding of the ECM composition found in normal and diseased tissues has recently improved with the aid of mass spectrometry-based proteomics (Byron et al., 2013). This is highly instructive for designing well-defined matrices that replace the ubiquitous use of Matrigel and provide more physiologically relevant microenvironment in that tissue-specific functions are dictated by the compositional and structural features of ECM (Bonnans et al., 2014; Magno et al., 2020). Ideally, defining key ECM components that serve as surrogates for the full matrix in supporting organoid growth would reduce the complexity of organoid culture. Alternatively, some of simplistic synthetic peptides derived from ECM components such as RGD can exert biological activity corresponding to those of the full proteins (Melkoumian et al., 2010; Deng et al., 2013; Hernandez-Gordillo et al., 2020). An early report on synthetic peptide-incorporated scaffolds demonstrated that surfaces made of acrylate conjugated to vitronectin-originated peptide supported self-renewal of human embryonic stem cells in a level similar to that of Matrigel which contains the full vitronectin (Melkoumian et al., 2010). However, the incorporation of single adhesive ligands may not be sufficient to induce proper organ morphogenesis, because native tissue has the complexity with adhesive ligands being present in different ECM proteins where each ECM displays multiple ligands to mediate distinct cell signalling pathways (Bonnans et al., 2014; Madl et al., 2018). Thus, further elaborate and systematic studies are needed to identify key microenvironmental parameters including ligand types and density that mainly govern stem cell behaviour and tissue-specific morphogenesis.

Aside from providing adhesive motifs, cell-relevant aspects of ECM mechanics including stiffness, viscoelasticity, and degradability should be considered. Stiffness of scaffolds can direct the differentiation of stem cells towards different lineages (Engler et al., 2006). In addition, a stress-relaxation of 3D scaffold is another key characteristic to regulate cell-ECM interactions. For example, alginate gels with a fast stress relaxing behaviour facilitated osteogenic differentiation of mesenchymal stem cells (MSCs), which was mediated through integrin-based adhesion, actomyosin contractility, local clustering of adhesion ligands, and nuclear localization of YAP (Chaudhuri et al., 2016). Moreover, the importance of dynamic microenvironments is highlighted by the fact that the formation and differentiation of epithelial organoids were highly dependent on the gradual matrix softening by degradation over time (Gjorevski et al., 2016). To generate such mechanically sophisticated scaffolds with tunable properties, dynamic polymer chemistries can be employed by finely tuning the ratio of both covalent and non-covalent, weak crosslinks and incorporating enzymatically, hydrolytically, or photochemically degradable motifs (Kharkar et al., 2013; Torgersen et al., 2013).

Furthermore, existing tissue engineering methodologies, such as microfluidics and bioprinting, enable the recapitulation of organism-level parameters including morphogen gradient, movement, fluid, and oxygen level that are required for morphogenesis and tissue homeostasis but often absent in traditional culture system (Homan et al., 2019; Jalili-Firoozinezhad et al., 2019; Shin and Kim, 2022). Particularly, computational modelling is helpful to predict the diffusion profile of soluble molecules, gas, and flow rate within the engineered matrices and microfluidic devices (Jin et al., 2018; Achberger et al., 2019; Homan et al., 2019; Manfrin et al., 2019). Indeed, in the recent study about engineering signaling centers for spatially controlled patterning of 2D hiPSC colonies, computational simulation was mainly utilized to predict both spatiotemporal concentration of BMP4 diffused from a source side and the local percentage of pSMAD1+ cells in response to BMP4 gradients (Manfrin et al., 2019). Importantly, diffusion experiments with Texas Red-labeled dextran and pSMAD staining of hiPSCs supported the simulated behaviours. Taking advantages of microfabrication-based devices, the integration of multiple organoids in one platform provides insights into crosstalk and interplay between different types of tissues/organs (Imura et al., 2010; Miller and Shuler, 2016; Ronaldson-Bouchard et al., 2022). Despite their efficacy at achieving spatiotemporal control over external cues, there is still room for improvement to overcome several hurdles such as the complexity in design and handling and to support long-term growth and maintenance of the organoids.

Compared with other epithelial organoids, ESC or PSC-derived skin organoids were more recently developed (Lee J. et al., 2018; Lee et al., 2020), and such bioengineering approaches have not been explored for the generation of skin organoids. The preceding approaches to produce multilayered skin have focused on the coculture of dissociated epidermal and dermal cells from newborn skin (Lei et al., 2017; Weber et al., 2019). The mixture of progenitor cells self-assembled into skin organoids containing placodes, dermal condensates, and hair peg-like structures. Furthermore, recent studies from Dr. Koehler’s research group showed the generation of hiPSC-derived skin organoids cultured in medium suspension containing dissolved Matrigel (Lee et al., 2020). Despite the impressive multicellular development and the generation of skin appendages including hair follicles, this system still suffers from the same shortcomings of other organoid culture systems such as uncontrolled spontaneous cellular organization and reliance on Matrigel. The generated skin organoids displayed an inside-out morphology where the dermis layer entirely covered the epidermal layer. Advanced engineering and biomaterials technologies we discussed above can be a breakthrough to overcome the limitations of current systems and to generate anatomically relevant skin organoids by providing precise spatiotemporal control over biochemical and physical cues.

In summary, current organoid culture systems still suffer from heterogeneity in their shape and variability in their functionality. The combination of well-defined biomaterials and tissue engineering techniques will continue to allow robust and reproducible generation of organoids by mimicking dynamic characteristics of microenvironments during tissue development. The ability to generate physiologically-relevant organoid models will have a major impact on the establishment of human disease models, and their integration into the drug discovery pipeline, supplementing and/or replacing animal experiments in preclinical studies.
